# Narcissistic Personality and Its Relationship with Post-Traumatic Symptoms and Emotional Factors: Results of a Mediational Analysis Aimed at Personalizing Mental Health Treatment

**DOI:** 10.3390/bs12040091

**Published:** 2022-03-25

**Authors:** Casandra I. Montoro, Pablo de la Coba, María Moreno-Padilla, Carmen M. Galvez-Sánchez

**Affiliations:** Department of Psychology, University of Jaén, 23071 Jaén, Spain; pcoba@ujaen.es (P.d.l.C.); mmpadill@ujaen.es (M.M.-P.)

**Keywords:** narcissism, emotional regulation, intolerance to uncertainty, perceived stress, positive and negative affect, post-traumatic symptoms, resilience

## Abstract

Background: Narcissism is characterized by entitlement, grandiose fantasies and the need for admiration. This personality trait has been associated with both traumatic experiences and emotional problems. Most studies have only focused on narcissism in the context of childhood trauma and negative emotional factors. However, dimensions of grandiose narcissism such as authority have been linked to adaptive outcomes. Furthermore, narcissism might not be linked only to negative childhood experiences; it may also be associated with the presence of post-traumatic symptoms. Therefore, the present study aimed to assess the associations between narcissism and the frequency and severity of post-traumatic symptoms and emotional factors (resilience capacity, emotional regulation, positive and negative affect, intolerance of uncertainty and perceived stress), as well as the possible mediational role of the latter in the relationship between narcissism and post-traumatic symptoms. Method: A total of 115 healthy young psychology undergraduates and their relatives, aged from 18 to 40 years, were asked to complete a set of questionnaires to evaluate the aforementioned variables. Results: The results showed that most of the grandiose narcissism dimensions were positively related to emotional adaptive outcomes, except exploitativeness and entitlement. The negative associations observed between the frequency and severity of post-traumatic symptoms and narcissism (self-sufficiency) were mediated by affect and resilience, which were in turn positively associated with the majority of the narcissism dimensions. Both positive affect and resilience were important factors mediating the association between grandiose narcissism and post-traumatic symptoms. Conclusions: Our findings reaffirm the need to assess not only desirable personality traits, but also ones that are not initially desirable, before pathologizing them. This consideration may be essential to achieve a personalized approach to the prevention of mental health problems, and promotion of positive emotions, in the general population.

## 1. Introduction

Narcissism is a personality trait characterized by a grandiose self-concept, as well as by behaviors intended to maintain this self-concept in the face of reality [1]. Core traits of narcissism include entitlement, grandiose fantasies and the need for admiration [2]. Both “normal” and “pathological” narcissism have been related to the experience of violence, suggesting that narcissism is an important risk factor for self-inflicted aggression and psychological abuse [3]. Pathological narcissism, obsessive compulsive and borderline personality are considered the most prevalent personality disorders in the general population [4]. Elevated narcissism often sets up a cascade of interpersonal and mental health challenges [5,6,7,8], and has even been associated with criminal behavior [9].

Several theories have emerged to obtain insight into the prevention and treatment of behavioral problems associated with narcissism. Most recent theories have focused on the link between narcissism and negative childhood experiences, such as physical or sexual violence, neglect, or rejection [10,11]. The emergence and development of narcissistic traits, such as seeking excessive admiration from others, feelings of grandiosity and interpersonal competitiveness, have mostly been related to traumatic experiences in childhood [12,13,14]. However, some studies have also pointed out that narcissistic characteristics may not only arise from childhood environments characterized by neglect/abuse, but also from environments in which a child is sheltered or overly praised [11,14,15]. Both of those situations foster an unrealistic image of the child (devaluation and idealization, respectively [16]). It has also been proposed that underlying vulnerability to post-traumatic stress may partially stem from a narcissistic personality disorder or narcissistic personality traits [17,18].

Unfortunately, most studies conducted to date have failed to exclusively explore the negative factors associated with narcissism, which are difficult to deal with once they become firmly entrenched and expressed in the individual’s behavior (e.g., negative affect, concerns about humiliation or lack of forgiveness for public transgressions [19]). Few studies have analyzed the relationship between narcissism and healthy emotional factors. Typically, narcissism has been related to difficulties in emotion regulation [20,21,22], simultaneous with resilience capacity [23]. However, the reported relations seem to depend on the type of narcissism [24]. It is important to note that narcissism can be differentiated into grandiose and vulnerable types [25,26]. Individuals with vulnerable narcissism tend to be anxious, defensive and avoidant, while grandiose narcissists are extraverted and self-satisfied, with a high propensity to strive for feelings of uniqueness and supremacy, and to devaluate others [25,26]. Sękowski et al. (2021) [23] aimed to elucidate the association between facets of narcissism and resilience, and found that grandiose narcissism was strongly associated with adaptive capacity, while vulnerable narcissism was associated with less resilience capacity. Loeffler et al. (2020) [24] observed a high tendency for the use of the maladaptive regulation strategy of suppression by individuals scoring highly for vulnerable narcissism compared to those scoring highly for grandiose narcissism. Similarly, other authors have differentiated between adaptive narcissism, which is psychologically healthy and related to resilience [27], and maladaptive narcissism, which is associated with entitlement and negative affect [2].

The same disparity in results can be seen with respect to the association between narcissism and perceived stress. Papageorgiou et al. (2019) [28] reported that subclinical narcissism was a predictor of lower perceived stress, while Coleman et al. (2019) [29] found that grandiosity and vulnerability narcissistic traits were related to altered stress reactivity. The differential influence on stress reactivity has been suggested to depend on the association of resilience capacity with the grandiose narcissism trait [29]. In support of this, Kajonius and Björkman [30] described a strong positive relationship between vulnerable narcissism and perceived stress, while grandiose narcissism showed a weak negative relationship with perceived stress.

Considering the two types of narcissism, it seems that grandiose narcissism might be a protective factor against emotional problems. This is attributable to the fact that, although in the long-term narcissism is characterized by negative interpersonal functioning, in the short term it is characterized by positive intrapersonal functioning (e.g., high self-esteem) [31]. Nonetheless, according to the multidimensional nature of grandiose narcissism, its effects on well-being outcomes are likely to depend on the dimensions; leadership/authority has generally been linked to adaptive outcomes, whereas entitlement and exploitativeness have been associated with maladaptive outcomes [32,33].

Overall, there is conflicting research regarding narcissism. Almost all studies conducted to date mostly focused on the well-established association between narcissism and childhood traumatic experiences. However, the plausible impact of narcissistic traits on psychological adjustment in relation to other traumatic experiences, and therefore the presence of post-traumatic symptoms, has rarely been [17,18]. Moreover, despite the proven utility of the transdiagnostic approach in the treatment of mental health problems [5,34,35,36], and the mixed results regarding the relationship between the two types of narcissism (Vulnerable vs. Grandiose) and mental health adjustment, few studies have focused on their individual constituents, especially with respect to grandiose narcissism dimensions, the effects of which on health outcomes and self-regulation seem to be mediated by a positive outlook [32,33].

Given the aforementioned relationships between narcissism and emotional factors, and the conflicting research regarding narcissism, the present study aimed to: (1) explore the association between narcissism and the frequency and severity of post-traumatic symptoms; (2) analyze the association between narcissism and emotional factors such as resilience capacity, emotional regulation, positive and negative affect, perceived stress and intolerance of uncertainty; and (3) study the possible mediational role of emotional factors on the relationship between the various narcissistic personality traits and the frequency and severity of post-traumatic symptoms. Additionally, owing to the reported mediational role of resilience in the effect of grandiose narcissism on stress reactivity [29], an exploratory mediational analysis of the different narcissism dimensions, perceived stress and resilience was conducted. Studies analyzing the different traits of grandiose narcissism and their relation to emotional factors, such as the present one, might be especially important for: (1) proper management and promotion of the emotional factors that may lead to a better psychological adjustment in people prone to mental health impairment due to binomial narcissism-traumatic experiences, (2) providing greater insight into the interpersonal and mental health challenges that narcissism entails, and (3) clarifying the benefits that some narcissism dimensions can have on emotional/mental health outcomes, including possible protection against the development of post-traumatic symptoms.

## 2. Materials and Methods

### 2.1. Participants

A total of 115 non-clinical psychological undergraduated university of Jaén students and their relatives (of whom 94 were female and 20 were male), aged from 18 to 40 years (M = 22.17 years, SD = 4.64), were asked to fill in a set of questionnaires. All of the participants voluntarily took part in the study and provided their data to the researchers. The participation agreement was made explicit by the signing of an informed consent form, and all participants received course credit for taking part. The exclusion criteria included suffering from any mental condition and the consumption of certain substances (e.g., anxiolytics, antidepressants, hypnotics, benzodiazepines or illegal psychoactive drugs).

### 2.2. Procedure and Psychological Measures

An identification code was generated to maintain the anonymity of the participants, who were asked to complete all of the below-described self-report questionnaires. The questionnaire order was counterbalanced between participants. All questionnaires were filled out in a single session. The study protocol was approved by the Bioethics Committee of the University of Jaén (DIC.20/7.PRY).

The Narcissistic Personality Inventory (NPI) was developed by Raskin and Terry (1988) [37] and translated into Spanish by García and Cortés (1998) [38]. This scale measures the narcissistic personality trait through 40 items and 7 sub-scales: (1) Leadership/Authority, i.e., the ability to control and influence others, (2) Exhibitionism, i.e., the tendency to be the center of attention, (3) Superiority, i.e., the belief (and consequently behavior) of being better than others, (4) Entitlement, i.e., the feeling of having more rights than others and deserving special treatment, (5) Exploitativeness, i.e., the tendency to exploit others without empathizing with their emotions, needs or interests, (6) Self-sufficiency, i.e., the belief that one achieves everything on one’s own, and (7) Vanity, i.e., excessive pride in or admiration of one’s own appearance or accomplishments. The items are responded to via a forced choice between narcissistic and non-narcissistic alternative options (e.g., “I really like to be the center of attention” vs. “It makes me uncomfortable to be the center of attention” [39]). The Cronbach’s α (internal consistency) of the Spanish version of the scale is 0.72 [38]. The NPI has been used as the main measure for grandiose narcissism [31,37].

The Connor–Davidson Resilience Scale (CD-RISC) was developed by Connor and Davidson (2003) [40] and translated into Spanish by Bobes et al. (2001) [41]. This scale consists of 25 items spread among five factors measuring the ability to overcome negative experiences and emerge stronger. The five factors are: personal competence, high standards, and tenacity (Persistence); trust in one’s instincts, tolerance to negative affect and the character-building effects of stress (Purpose); positive acceptance of change and secure relationships (Adaptability); Control; and Spirituality. Scores range between 0 (Not at all true) and 4 (True nearly all the time) on all subscale items. The total scale score is in the range of 0 to 100, with higher scores indicating a higher level of resilience. The Cronbach’s α of the Spanish version of the scale is 0.86 [42].

The Davidson Trauma Scale (DTS) is a self-rating scale used for diagnosing and measuring symptom severity and treatment outcomes in post-traumatic stress disorder which was developed by Davidson et al. (1997) [43] and translated into Spanish by Rodríguez-Rey et al. (2016) [44]. This instrument assesses the frequency and severity of symptoms of post-traumatic stress disorder in patients who have experienced trauma. It is composed of 17 items scored using a five-point Likert scale (0 = never or nothing, 4 = daily or extreme). The Cronbach’s α of the total scale is 0.99, ranging between 0.97 for the Frequency scale and 0.98 for the Severity scale [45].

The original Perceived Stress Scale (PSS) was developed by Cohen et al. (1983) [46] and translated into Spanish by Remor and Carrobles (2001) [47]. This self-report instrument assesses the level of perceived stress during the last month. It comprises 14 items and used a five-point scale response format (0 = never, 4 = very often). Total scores range from 0 to 56, and higher scores reflect higher levels of perceived stress. The Cronbach’s α for the PSS ranges between 0.82 and 0.85 [48].

The Intolerance of Uncertainty Scale (IUS) was developed by Freeston et al. (1994) [49] and translated into Spanish by González Rodríguez et al. (2006) [50]. The IUS is a five-point Likert scale with 27 items ranging from 1 (not at all characteristic of me) to 5 (entirely characteristic of me) and subdivided into two main factors: inhibition eliciting uncertainty (IGI, 16 items) and uncertainty manifesting as confusion and unexpectedness (IDI, 11 items). Smith et al., 1995 [51] reported a Cronbach’s α of 0.91 for the IUS (0.93 for IGI and 0.89 for IDI).

The Positive and Negative Affect Scale (PANAS) was developed by Watson et al. (1988) [52]. The 20 PANAS items respond to a five-point Likert scale ranging from 0 (nothing or almost nothing) to 4 (very much); it is subdivided into two main factors, which correlate negatively and independently: (1) positive affect, which includes affective states with positive valence, such as joy, enthusiasm, and a positive mood, and refers to a pleasant state of mind characterized by motivation, energy, greater desire for affiliation, a sense of belonging and life satisfaction; and (2) negative affect, which includes negative states of mind such as sadness, fear, anxiety or anger, and refers to a subjective discomfort that can act as a latent factor promoting the depression [52]. Scores range from 20 to 100. The positive and negative affect factors have Cronbach’s α values of 0.85 and 0.89, respectively [53].

The Emotional Regulation Questionnaire (ERQ) was developed by Gross and John (2003) [54] and translated into Spanish by Cabello et al. (2013) [55]. The ERQ is a 10-item instrument scored using a seven-point Likert- scale that ranges from 0 (totally disagree) to 4 (totally agree). It assesses two factors: emotional suppression (inhibition of expression of emotional behaviors with no obvious decrease in the intensity of the negative emotion) and cognitive reappraisal (ability to construct a new and adaptive meaning to mitigate the negative emotional impact of a specific situation). Total scores on the scale are in the range of 0 to 100, and higher scores indicate a higher level of resilience. The emotional suppression and cognitive reappraisal factors have Cronbach’s α values of 0.82 and 0.77, respectively [56].

### 2.3. Data Analysis

In order to determine the optimal sample size for the associations, the G*Power 3.1.7 program was used [57]. Assuming an effect size of 0.50, alpha level of 0.05 and beta error of 20%, a sample size of 21 participants was optimal. In a first step, a descriptive analysis of all of the narcissism and emotional dimensions was conducted. Relationships between narcissistic personality dimensions and the other self-reported emotional variables were quantified using Pearson correlations, followed by multiple regression analysis. The seven narcissistic personality dimensions were entered simultaneously as predictors; the rest of the psychological variables (in a separate analysis) were the dependent variables. Finally, in order to explore the relationship between narcissism and post-traumatic symptoms more deeply, including the possible mediating role of the emotional variables, mediational analyses were conducted. Significance was set at *p* ≤ 0.05. Mediation analyses were then performed with the PROCESS macro for SPSS, to assess the significance of partial mediation effects based on K. J. Preacher’s algorithms [58,59]. This test statistically compares the difference between the direct effects of a predictor variable and indirect effects occurring through a mediating variable. Furthermore, to increase the robustness of the results, confidence intervals (CIs) were generated through bootstrapping effect estimation techniques. For significant mediating effects, the limits of the CI should do include the 0 value. A total of 5000 bootstrap resamples were used to generate bias-corrected 95% CIs for the indirect effect.

## 3. Results

### 3.1. Mean and Standard Deviation Scores on of the Different Narcissism Sub-Scales and Emotional Variables

Table 1 shows the mean and standard deviation scores on the different narcissism sub-scales and emotional variables evaluated in the present sample.

### 3.2. Associations between Narcissistic Personality Dimensions and Post-Traumatic Symptoms

The frequency of post-traumatic symptoms was positively associated with entitlement (r = 0.186, *p* = 0.047) and negatively associated with self-sufficiency (r = −0.364, *p* ≤ 0.001) and vanity (r = −0.215, *p* = 0.021), while post-traumatic symptom severity was negatively associated with self-sufficiency (r = −0.281, *p* = 0.002).

### 3.3. Associations between Narcissistic Personality Dimensions and Positive and Negative Affect

Positive affect was positively associated with authority (r = 0.391, *p* ≤ 0.001), exhibitionism (r = 0.304, *p* = 0.001), superiority (r = 0.265, *p* = 0.004), self-sufficiency (r = 0.279, *p* = 0.003) and vanity (r = 0.537, *p* ≤ 0.001). Negative affect was negatively associated with self-sufficiency (r = −0.233, *p* = 0.012) and vanity (r = −0.208, *p* = 0.026).

### 3.4. Associations among Narcissistic Personality Dimensions, Perceived Stress and Emotional Regulation

Perceived stress was negatively associated with self-sufficiency (r = −0.409, *p* ≤ 0.001) and vanity (r = −0.317, *p* = 0.001). Cognitive reappraisal was positively with authority (r = 0.201, *p* = 0.031) and self-sufficiency (r = 0.195, *p* = 0.037) while emotional suppression was negatively associated with superiority (r = −0.224, *p* = 0.016) and vanity (r = −0.236, *p* = 0.011), and positively associated with entitlement (r = 0.200, *p* = 0.032).

### 3.5. Associations between Narcissistic Personality Dimensions and Resilience

Table 2 shows the Pearson correlations between narcissistic personality dimensions and resilience sub-scales. For authority, exhibitionism, self-sufficiency and vanity, positive significant associations with all of the resilience sub-scales (except spirituality) were observed. Exploitativeness was negatively associated with spirituality. No significant associations were observed for the superiority and entitlement dimensions.

### 3.6. Associations between Narcissistic Personality Dimensions and Intolerance of Uncertainty

Intolerance of uncertainty was negatively associated with authority (r = −0.202, *p* = 0.030), superiority (r = −0.246, *p* = 0.008), self-sufficiency (r = −0.302, *p* = 0.001) and vanity (r = −0.263, *p* = 0.004), and positively associated with entitlement (r = 0.235, *p* = 0.011).

### 3.7. Regression Analysis

Table 3 shows the results of multiple regression analysis of the predictors of the outcomes of interest. Most of the significant associations in the correlation analysis remained significant in the regression analysis.

Regarding post-traumatic symptoms, and according to the correlation analysis, self-sufficiency negatively predicted both the frequency and severity of post-traumatic symptoms. Vanity also negatively predicted the frequency of post-traumatic symptoms. No significant association between entitlement and the frequency of post-traumatic symptoms was observed.

Regarding the resilience variables, authority positively predicted persistence, control, adaptability, purpose and the total resilience score. Exhibitionism positively predicted persistence, control and the total resilience score. Exploitativeness negatively predicted persistence, spirituality and the total resilience score. Self-sufficiency positively predicted persistence, adaptability, purpose and the total resilience score. Vanity positively predicted the control resilience sub-scale score.

Positive affect was positively predicted by authority and vanity. There were no significant predictors of negative affect. Emotional suppression was negatively predicted by superiority and vanity, and positively predicted by entitlement. Finally, intolerance of uncertainty was negatively predicted by authority, superiority, self-sufficiency and vanity, and positively predicted by entitlement.

### 3.8. Mediational Analyses

The mediation analyses revealed that persistence (resilience), purpose (resilience), total resilience and positive affect were significant mediators of the relationship between the frequency of post-traumatic symptoms and exhibitionism (narcissism), while persistence (resilience), adaptability (resilience), purpose (resilience), total resilience, positive affect, negative affect and intolerance of uncertainty were significant mediators of the relationship between the frequency of post-traumatic symptoms and self-sufficiency (narcissism).

**Figure 1:**
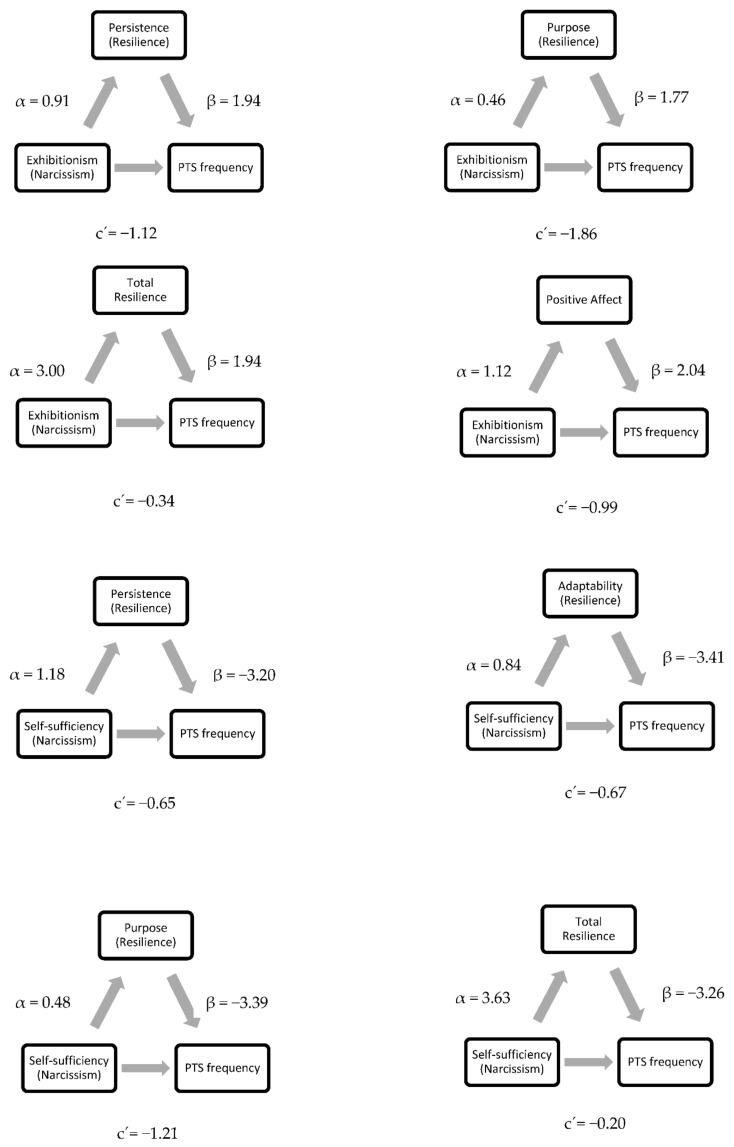

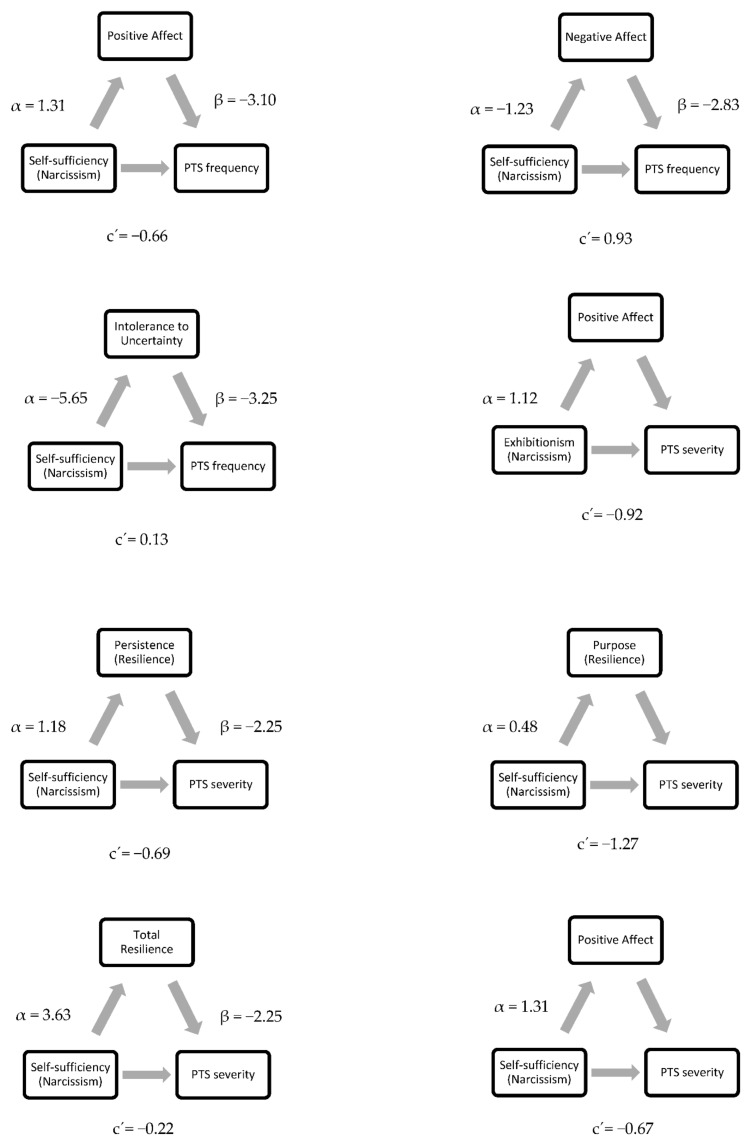

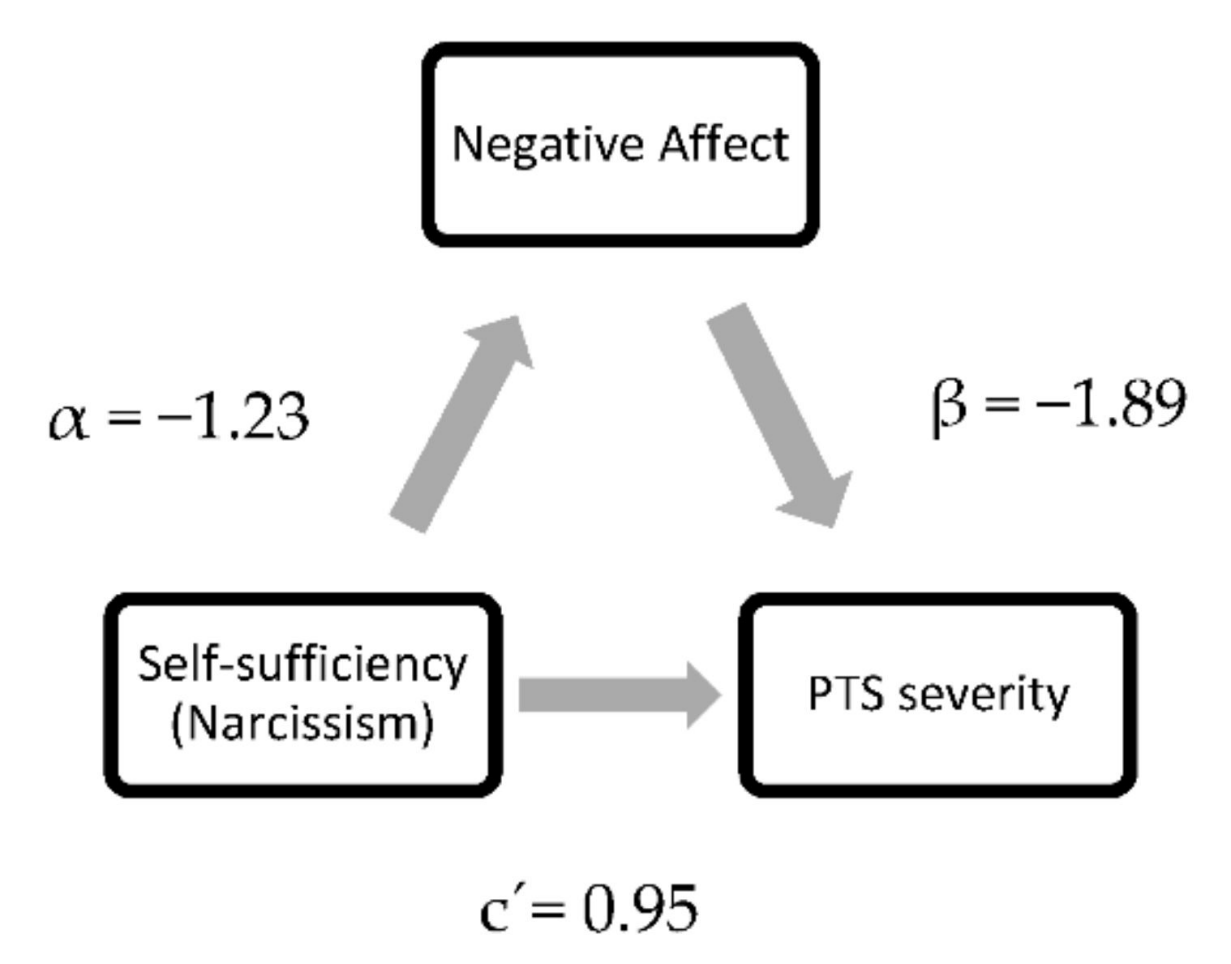
Statistical diagrams of partial mediation effects of positive and Negative Affect, resilience and Intolerance to Uncertainty on the relation between Narcissism and Post-traumatic symptoms (PTS; Frequency and Severity).

Positive affect was a significant mediator of the relationship between post-traumatic symptom severity and exhibitionism (narcissism). In addition, persistence (resilience), purpose (resilience), total resilience, positive affect, and negative affect were significant mediators of post-traumatic symptom severity and self-sufficiency (narcissism). More details are provided in Table 4 and Figure 1.

**Figure 2:**
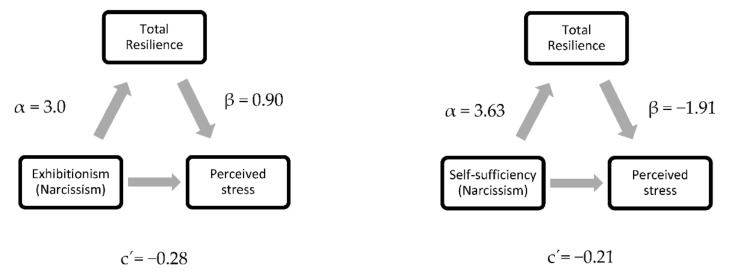

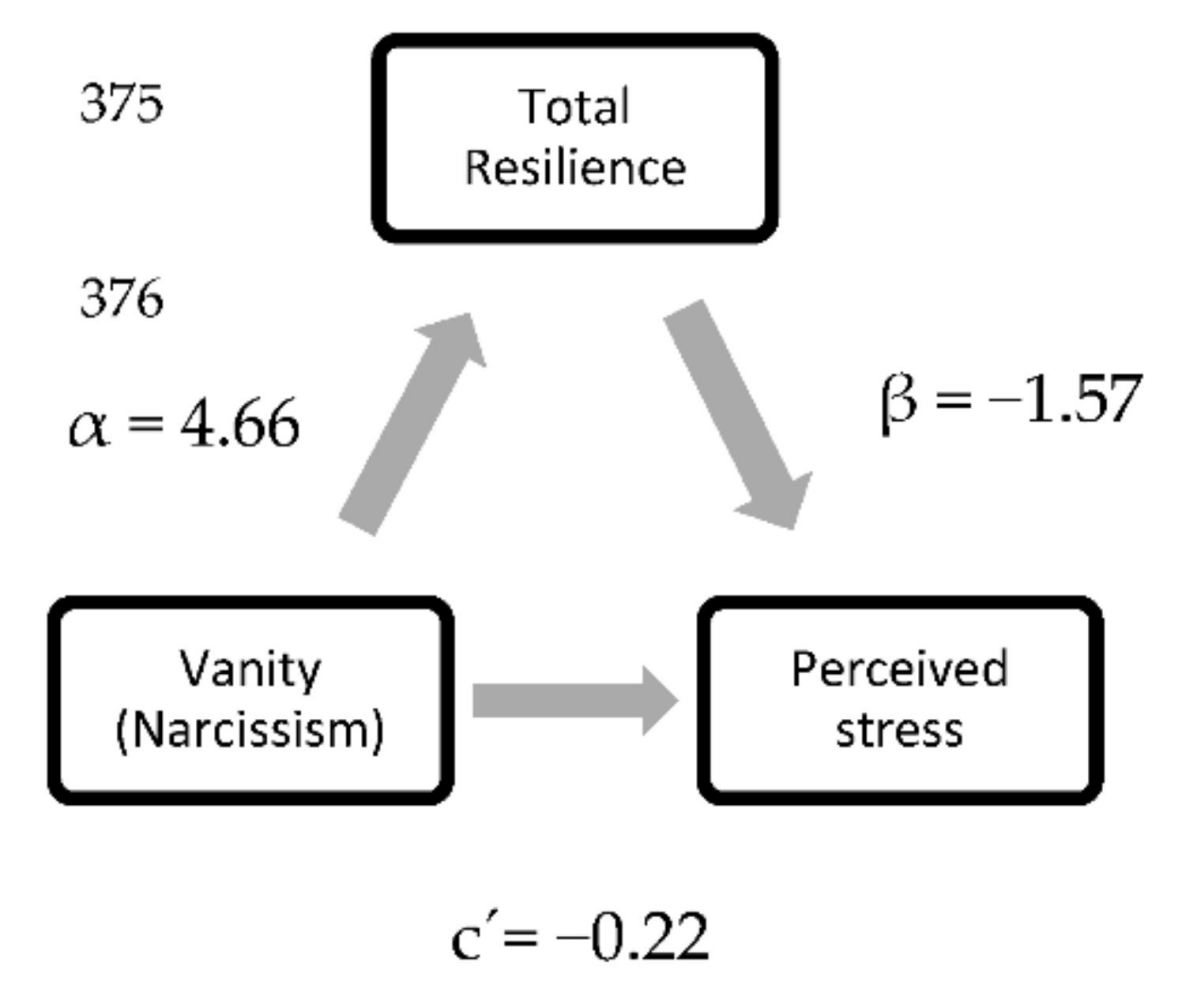
Statistical diagrams of partial mediation effects of Resilience on the relation between Narcissism and Perceived Stress.

Concerning the additional objective of the current research, Table 5 and Figure 2 show that total resilience was a significant mediator in the relationship between narcissistic traits (exhibitionism, self-sufficiency and vanity) and perceived stress.

## 4. Discussion

This study aimed to assess the association between narcissism and the frequency and severity of post-traumatic symptoms, as well as the possible mediational role of emotional factors (resilience capacity, emotional regulation, positive and negative affect, intolerance of uncertainty and perceived stress). Consistent with our predictions, correlation and regression analyses indicated significant associations between narcissism and all of the emotional factors and post-traumatic symptoms dimensions evaluated. Specifically, the frequency of post-traumatic symptoms was positively related to entitlement, and negatively to vanity and self-sufficiency. This latter negative association was also found for post-traumatic symptom severity. Despite the fact that narcissism has mostly been studied in association with negative experiences in childhood, and has been proposed to arise from such experiences [60,61,62], its association with the severity and frequency of post-traumatic symptoms in the present study confirms the suggestion by Levi and Bachar (2019) [17] and Simon et al. (2002) [18] of an association between narcissism and post-traumatic symptoms that goes beyond childhood.

However, at first glance, the associations of lower post-traumatic symptom frequency and severity with greater vanity and self-sufficiency may seem incongruent with the proposed link between vulnerability to trauma and narcissistic personality traits [17,18]. Nonetheless, and with respect to self-sufficiency, the analyses conducted in the present study demonstrated mediational roles of affect and resilience in this relationship, with might explain the lower frequency and severity of post-traumatic symptoms associated with this narcissism dimension.

Moreover, vulnerability to post-traumatic symptoms might depend mainly on the entitlement trait which, among all of the narcissistic traits, was the only one related to worse post-traumatic outcomes. Regression analyses confirmed this pattern of associations, suggesting that vanity and self-sufficiency might protect against post-traumatic symptoms in some individuals, while entitlement would have a detrimental effect. Against this background, among all the grandiose narcissism dimensions, entitlement and exploitativeness have been proposed to constitute the core of narcissism [32,63], and mainly reflect maladaptive narcissism and a general tendency toward antagonism [5,6,7,8,32,33].

Regarding positive and negative affect, positive associations were observed between the majority of the grandiose narcissistic traits and positive affect, whereas the associations were negative for negative affect. Lower scores for the self-sufficiency and vanity traits were related to greater negative affect. However, while the associations between positive affect and vanity were confirmed by the regression analyses, those for negative affect were not. Previous literature has demonstrated a positive relationship between vulnerable narcissism and explicit negative affect [8,64]. However, the present findings indicated the opposite, where grandiose narcissism seemed to increase positive, and decrease negative, affect. In support of our findings, grandiose narcissism has been related to high-approach positive affect following provocation [65]. Hence, positive affect in individuals exhibiting grandiose narcissism might be a coping strategy promoting the accomplishment of goals [65].

Expanding previous research findings [28], grandiose narcissism, and specifically the dimensions self-sufficiency and vanity, showed a negative relationship with perceived stress in this study. Moreover, positive associations were found among cognitive reappraisal, authority and self-sufficiency, and negative ones among emotional suppression, superiority and vanity. Emotional suppression was also positively associated with entitlement. Our findings do not support a specific relationship between difficulties with emotional regulation and grandiose narcissism [24]. Surprisingly, entitlement was inversely associated with greater emotional suppression, and this association was confirmed by regression analyses. Given these results, it is reasonable to speculate that the negative/maladaptive connotation of entitlement [33] is likely to be a consequence of its relation with emotional suppression. Emotional suppression strategies are related to a type of avoidance of expressing emotional behaviors, as part of a larger defense mechanism that leads to relief in the short-term but has harmful long-term consequences. It has been demonstrated that while people who usually suppress emotions avoid suffering in the short term, they are subsequently faced with greater discomfort [66] and are more predisposed to mental health problems [67,68].

Similarly, most of the narcissism dimensions (authority, exhibitionism, self-sufficiency and vanity) were positively associated with all of the resilience sub-scales except spirituality, persistence and total resilience (the two last results were from the regression analyses), which were negatively associated with exploitativeness. The established strong association between grandiose narcissism and the adaptive capacity of resilience [23] was also confirmed by the present study. Finally, greater intolerance of uncertainty was associated with less pronounced narcissism characteristics in general, but this was especially true for entitlement. These findings confirm that entitlement and exploitativeness are key elements of maladaptive narcissism [32,33].

Concerning mediational analyses, the main findings were that greater positive affect and lower negative affect were significant mediators of the relationship between self-sufficiency (positive affect and negative affect) and post-traumatic symptom frequency and severity, confirming the protective role of positive affect in general health [69,70,71] and the need to enhance emotional education, especially in young populations. In fact, in previous studies, positive affect was proposed as a mediator of the forgiveness-health relationship [70]. Thus, it is possible that positive affect could serve as an important therapeutic target in trauma treatment, given its relationships with forgiveness and general health.

Moreover, resilience (especially the purpose and persistence dimensions) was also a significant mediator of the association between post-traumatic symptoms and self-sufficiency (post-traumatic symptom frequency and severity). Self-enhancement, which involves self-sufficiency, is a dimension of purpose previously linked to resilience [72,73]. Although some authors agree with the traditional idea that mental health requires realistic appraisal and acceptance of personal limitations and negative characteristics [72,73], other researchers argue that unrealistic or overly positive biases in favor of the self, such as self-enhancement, can be adaptive and promote well-being [74]. Likewise, trait self-enhancement has been associated with personal benefits, such as high self-esteem [74,75]. At this point, it is further important to note that although no direct associations were initially found between exhibitionism and the frequency and severity of post-traumatic symptoms in this study, when resilience and positive affect were included as mediating factors, indirect associations arose.

It is well-known that resilience is associated with a range of positive personal attitudes and behaviors [76]. By combining resilience resources with protective factors, individuals perform better and can remain healthy even under high pressure [77]. Resilience has been proposed as a key factor in the adaptation to stressful or traumatic events [78]. Congruent with this, our findings suggest that positive affect and resilience may promote coping with pressure and the negative consequences of trauma, given that they are central features in the explanation of post-traumatic symptoms based on the adaptive role of grandiose narcissistic traits.

Additionally, our findings supported a mediating role for total resilience in the association between grandiose narcissism and stress [29]. However, not all grandiose narcissism dimensions were significant in terms of this mediational result, which was limited to the exhibitionism, self-sufficiency and vanity dimensions; specifically, the findings suggest a mediational role of total resilience in the relationships between these specific narcissism dimensions and stress, but not narcissism as a whole.

Finally, intolerance of uncertainty was also a significant mediator of the association between post-traumatic symptom frequency and self-sufficiency (narcissism). The mediational model showed that greater self-sufficiency led to milder post-traumatic symptoms, as mediated by lower intolerance of uncertainty. Interest in the relationship between intolerance of uncertainty and emotions has rapidly increased over the last decade. In particular, the relationship between intolerance of uncertainty and the underlying ‘fear of the unknown’ has attracted attention, as has the role of intolerance of uncertainty in the development, maintenance, and treatment of a broad array of emotional disorders (e.g., generalized anxiety [79]). Furthermore, intolerance of uncertainty has been related to negative affect [80] and seems to play a crucial role in a transdiagnostic model relevant to clinical management [81]. Similar to emotional regulation, our results confirmed the importance of the management of intolerance of uncertainty in the transdiagnostic approach. However, given that the effect of intolerance of uncertainty was limited to the frequency of post-traumatic symptoms’ and self-sufficiency in this study, more research is required to draw firm conclusions.

Considering these mediational results, it is plausible that promoting certain facets of adaptive narcissism could be an appropriate strategy for enhancing emotional regulation and stress management in some individuals. Preventive healthcare should also be promoted to make individuals and communities more resilient, and to enhance positive affect. Furthermore, the scope of psychological interventions could be expanded such that they aim not only to modify personality traits that have traditionally been considered pathologic, but also to explore their benefits for emotional health and promote them. It seems that certain features of narcissism, i.e., those related to a positive orientation toward various life domains, including the self, may be crucial for psychological adjustment to adversity. Relatedly, emotional education is crucial for improving mental and physical health [82,83]. In line with the above and its known effectiveness, the transdiagnostic perspective on psychological inflexibility and emotional dysregulation is increasing in popularity [84]. Considering grandiose narcissistic traits (and their differential effects [adaptive vs. maladaptive] on emotional health outcomes) as part of the transdiagnostic perspective may be important for personalized behavior management, which has been demonstrated to be essential for mood regulation as an alternative to medications [85]. Nonetheless, the applications of the present study’s mentioned results should be considered with caution due to the non-casual connections nature of the study.

In this sense, the main limitations of our study were its cross-sectional design, which does not allow for the establishment of causal associations, and the no correction for Type I errors. Moreover, the analysis was based on self-reported measures, which could be sensitive to biases such as participant mood [86]. Furthermore, the use of self-report instruments might lead to bias in terms of the influence of emotional states on the reportage of symptom impact and severity. In addition, given the apparent gender differences in narcissism [87], it would be advisable for future studies to include more males in their samples, to allow binomial analysis of narcissism in relation to traumatic experiences and emotional factors. However, exploiting gender differences was not an objective of the present study, strengths of which included the novelty of the theme and clinical relevance to personalized treatment. Furthermore, our large sample size allowed for the performance of a mediation analysis, which provides greater insight into the complex interrelation between predictors. Similar studies to the present one, but including various clinical populations instead of healthy subjects, might also be instructive.

Finally, it is important to notice that in the cultural context of Spain, and opposite to that which occurs in other cultural contexts (e.g., Thailand), there is a tendency on Cognitive Empathy but not Affective Empathy [88]. Although narcissism has mainly been associated with the lack of Affective Empathy [89,90] and Spanish culture is not characterized by showing a general high tendency of narcissism traits, Spaniards’ socialization is based on competitiveness and self-sufficiency, that is, narcissistic traits that are generally praised by the Spanish population. In fact, a recent study found that high narcissism in Spanish athletes was related to both the desire to win and the fear of losing (related to self-esteem and perceived competence) [91]. In this sense, the negative associations observed between the frequency and severity of post-traumatic symptoms and self-sufficiency may be explained by the cultural context. Therefore, more studies replicating the current results are needed, in order to establish if the positive or negative health influence of narcissism is mediated not only by the culture but also by the region and geography. A considerable amount of evidence suggests that sociocultural environment, regional and geography psychological differences do exist [92,93] 

## 5. Conclusions

To conclude, grandiose narcissism dimensions measured by the NPI [31,37] seem to be related to emotional adaptive outcomes, with the exception of exploitativeness and entitlement (related to lower levels of emotional reappraisal (such as persistence, spirituality and total resilience) and greater emotional suppression and intolerance of uncertainty, respectively). Moreover, the negative associations between the frequency and severity of post-traumatic symptoms and narcissism (self-sufficiency) seem to be mediated by affect and resilience, which are in turn positively associated with the majority of the narcissism dimensions. Both positive affect and resilience were potent mediators of the association between narcissism and post-traumatic symptoms. Our findings indicate that it is important to assess not only desirable personality traits, but also others that are not desirable at first glance, such as those related to narcissism, in each specific health context before pathologizing them. To sum up, our results demonstrate that narcissistic traits might be, in some cases, adequate for coping with post-traumatic symptoms; consideration of this may be necessary to achieve a personalized approach to the prevention and promotion of emotional/mental health in the general population.

## Figures and Tables

**Table 1:** Mean (M) and standard deviation (SD) scores of the different narcissism sub-scales and and emotional variables in the present sample.

Narcissism Sub-Scales and Emotional Variables	(N = 115)	
	M	SD
**Narcissism sub-scales**		
Authority	3.35	1.86
Exhibitionism	1.73	1.61
Superiority	2.28	1.30
Entitlement	1.85	1.45
Exploitativeness	1.59	1.16
Self-sufficiency	2.18	1.26
Vanity	1.01	.99
**Post-traumatic symptoms**		
Frequency	24.25	14.03
Severity	22.25	14.21
**Affect**		
Positive affect	22.18	6.09
Negative affect	20.25	7.05
**Perceived stress**	27.26	8.31
**Emotional regulation**		
Cognitive reappraisal	30.00	6.44
Emotional suppression	15.68	5.89
**Resilience sub-scales**		
Persistence	16.03	4.75
Control	18.03	4.79
Adaptability	14.46	4.09
Purpose	7.59	2.68
Spirituality	4.32	2.06
Total resilience	60.43	15.44
**Intolerance of uncertainty**	74.97	23.37

**Table 2:** Pearson correlations between narcissistic personality dimensions and resilience.

Resilience Subscales	Authority	Exhibitionism	Superiority	Entitlement	Exploitativeness	Self-Sufficiency	Vanity
**Persistence**	0.278 **	0.316 **	0.096	0.084	−0.074	0.320 **	0.314 **
**Control**	0.356 **	0.312 **	0.065	0.046	0.108	0.252 **	0.223 *
**Adaptability**	0.305 **	0.227 *	0.061	−0.016	0.042	0.262 **	0.276 **
**Purpose**	0.296 **	0.283 **	0.158	0.168	−0.016	0.234 *	0.306 **
**Spirituality**	−0.077	0.076	0.007	0.055	−0.217 *	0.084	0.051
**Total**	0.319 **	0.315 **	0.095	0.073	0.010	0.299 **	0.300 **

Note. * *p* ≤ 0.05; ** *p* ≤ 0.001.

**Table 3:** Narcissistic personality traits as predictors of the outcomes of interest: Results of multiple regression analysis. β = unstandardized beta.

β								*r*^2^ Adjusted
	Aut	Exh	Sup	En	Exp	Self	Van	
**PTS Severity**	−0.027	0.222	0.966	1.26	−0.019	−2.75 *	−2.41	0.065
**PTS Frequency**	0.091	0.497	0.582	1.66	−0.586	−3.49 **	−2.79 *	0.144
**Persistence**	0.554 *	0.804 *	−0.042	−0.240	−1.10 *	1.06 **	0.831	0.257
**Control**	0.714 *	0.740 *	−0.188	−0.410	−0.317	0.756	0.441 *	0.179
**Adaptability**	0.607 *	0.418	−0.146	−0.443	−0.453	0.623 *	0.743	0.164
**Purpose**	0.323 *	0.268	0.091	0.064	−0.454	0.389 *	0.496	0.175
**Spirituality**	−0.082	0.176	0.033	0.062	−0.420 *	0.205	0.024	0.022
**Total**	2.116 *	2.406 *	−0.252	−0.968	−2.734 *	3.035 *	2.526	0.230
**Positive affect**	0.824 *	0.346	0.491	−0.069	−0.402	0.710	2.60 **	0.370
**Negative affect**	0.021	0.293	−0.427	0.764	−0.373	−0.962	−1.40	0.059
**Perceived stress**	−0.490	0.496	−0.419	−0.037	0.685	−2.254 **	−2.028 *	0.192
**Cognitive reappraisal**	0.661	−0.189	0.349	0.383	−0.697	0.811	0.492	0.040
**Emotional suppression**	−0.471	−0.184	−0.883 *	1.362 **	0.031	0.567	−1.406 *	0.144
**Intolerance of uncertainty**	−2.89 *	2.499	−3.557 *	4.482 *	0.567	−3.382 *	−5.485 *	0.249

Note. Authority = Aut; Exhibitionism = Exh; Superiority = Sup; Entitlement = En; Exploitativeness = Exp; Self-sufficiency = Self; Vanity = Van; Post-traumatic symptoms = PTS; * p ≤ 0.05; ** *p* ≤ 0.001.

**Table 4:** Results of mediation analysis of the predictors of post-traumatic symptom frequency and severity.

Independent Variables	Mediator Variables	Effect	SE	*p*	Boot LLCI	Boot ULCI
PTS Frequency						
**Exhibitionism (narcissism)**	Persistence (Resilience)	−0.117	0.041	0.0178	−0.203	−0.046
	Purpose (Resilience)	−0.097	0.037	0.0296	−0.177	−0.035
	Total Resilience	−0.116	0.042	0.0188	−0.204	−0.043
	Positive affect	−0.128	0.039	0.0107	−0.208	−0.058
**Self-sufficiency (narcissism)**	Persistence (Resilience)	−0.069	0.035	0.0020	−0.146	−0.008
	Adaptability (Resilience)	−0.051	0.031	0.0009	−0.120	−0.001
	Purpose (Resilience)	−0.052	0.029	0.0008	−0.116	−0.004
	Total Resilience	−0.064	0.035	0.0016	−0.139	−0.004
	Positive affect	−0.078	0.040	0.0020	−0.169	−0.013
	Negative affect	−0.103	0.040	0.0018	−0.187	−0.027
	Intolerance of uncertainty	−0.065	0.038	0.0017	−0.149	−0.004
**PTS severity**						
**Exhibitionism (narcissism)**	Positive affect	−0.117	0.037	0.0442	−0.198	−0.051
**Self-sufficiency (narcissism)**	Persistence (Resilience)	−0.072	0.039	0.0356	−0.159	−0.009
	Purpose (Resilience)	−0.053	0.031	0.0184	−0.123	−0.006
	Total Resilience	−0.072	0.038	0.0336	−0.156	−0.008
	Positive affect	−0.078	0.039	0.0358	−0.165	−0.016
	Negative Affect	−0.104	0.041	0.0440	−0.190	−0.029

Note: Indirect effects are reported. SE = standard error. Boot = bootstrapping results with confidence intervals for the lower (LLCI) and upper limits (ULCI). PTS = post-traumatic symptoms. All coefficients are standardized.

**Table 5:** Results of mediation analysis of the predictors of perceived stress.

Independent Variables	Mediator Variables	Effect	SE	*p*	Boot LLCI	Boot ULCI
Perceived Stress						
Exhibitionism (narcissism)	Total resilience	−0.164	0.045	0.0470	−0.259	−0.082
Self-sufficiency (narcissism)	Total resilience	−0.114	0.043	0.0007	−0.208	−0.041
Vanity (narcissism)	Total resilience	−0.124	0.047	0.0319	−0.224	−0.042

Note: Indirect effects are reported. SE = standard error. Boot = bootstrapping results with confidence intervals for the lower (LLCI) and upper limits (ULCI). All coefficients are standardized.

## Data Availability

The authors claim that this manuscript describes an original research work which has not been preregistered. The data presented in this study are available on request from the corresponding authors. The data are not publicly available due to compliance with privacy laws.
